# Research on Energy Transmission Characteristics of Mechanical Vibrations in Steel Fiber-Reinforced Concrete

**DOI:** 10.3390/ma19091693

**Published:** 2026-04-22

**Authors:** Feilong Zhang, Chong Wang, Baosheng Xu, Liangqi Zhang

**Affiliations:** 1 College of Materials Science and Engineering, Chongqing University, Campus B, Chongqing 400044, China; zhangfeilong0608@163.com; 2 Detong Intelligent Technology Co., Ltd., Xuchang 461000, China; xbsjntn@163.com (B.X.); 17630829382@163.com (L.Z.)

**Keywords:** mechanical vibration, steel fiber-reinforced concrete, vibration intensity, velocity displacement, energy transmission behavior

## Abstract

The mixing process is a critical factor influencing the performance of concrete. As an effective method for enhancing mixing, vibratory stirring relies on the propagation characteristics of mechanical vibration within the concrete matrix. To investigate the propagation behavior of mechanical vibration in fresh steel fiber-reinforced concrete, a custom-developed mechanical vibration source and testing system was established. The results show that the vibration intensity attenuates to 50% at a distance of 5 cm from the source, to approximately 10% at 10 cm, and to less than 3% at 20 cm. A lower water-to-binder ratio facilitates the transmission of the vibration wave, while the presence of fibers and 0–5 mm coarse aggregates hinders vibration propagation. Based on these findings, an input–output energy conservation equation was developed to describe the transmission behavior of vibration energy. The numerical results were compared with experimentally measured vibration power and particle velocity displacement integrals, validating the effectiveness of the proposed energy conservation equation.

## 1. Introduction

As the most widely used construction material in modern engineering projects in terms of volume and application scope, the performance of cement concrete in its fresh state directly dictates the mechanical strength and durability of the hardened components [1]. With the development of infrastructure towards ultra-high-rise and large-span structures, more stringent requirements are imposed on the workability and microstructural homogeneity of cement-based materials during construction.

Traditional mixing processes primarily rely on macroscopic convection to achieve paste uniformity. However, due to the large specific surface area and high surface energy of cement, flocculation structures readily form, encapsulating free water. This leads to reduced workability, increased energy consumption, and microstructural defects. Microscopic observations indicate that even after conventional mixing, 10% to 30% of cement particles still exist in the form of agglomerates [2]. The internal pores of these agglomerates remain un-wetted, with particles in a “dry-mix” state, which not only wastes cementitious material but also limits the improvement of concrete performance. To address this, researchers have attempted to enhance the mixing process by optimizing blade installation angles [3], adjusting the arrangement of mixing arms [4], and increasing the rotational speed of mixing equipment [5].

However, these measures are essentially local optimizations within existing mixing modes and do not fundamentally resolve the issue of micro-scale cement particle agglomeration. To overcome this challenge, vibratory mixing technology emerged.

Currently, scholars worldwide have conducted extensive research on vibratory mixing technology and achieved a series of fruitful results, focusing on three main aspects:

First, comparative studies between vibratory mixing and traditional mixing processes. Zhao et al. [6] investigated the workability of cement-stabilized macadam prepared by traditional mixing and vibratory mixing, finding that vibratory mixing can enhance workability while reducing cement consumption and carbon emissions. Tian et al. [7] compared three processes—traditional mixing, vibratory mixing, and vibration cessation during mixing—and confirmed that vibratory mixing yields the most significant improvement in the uniformity of cement-stabilized macadam mixtures. Zheng et al. [8] prepared ultra-high performance concrete using both ordinary mixing and vibratory mixing techniques, demonstrating that vibratory mixing not only imparts a faster strength development rate but also results in superior 28-day compressive strength and splitting tensile strength compared to ordinary mixing. Zhang et al. [9], based on comparative experiments between traditional forced mixing and vibratory mixing, further confirmed the significant technical advantages of vibratory mixing in enhancing UHPC performance. Li et al. [10] compared vibratory mixing technology with conventional mixing methods and found that cement subjected to vibratory mixing exhibited a more uniform distribution of hydration products and a three-dimensional network structure, which effectively reduces drying and thermal shrinkage of cement while enhancing fatigue life.

Second, research on the strengthening effects of vibratory mixing on material properties. Wang et al. [11] investigated the influence of vibratory mixing on the performance of recycled aggregate cement-stabilized macadam, confirming that it significantly improves compressive strength as well as drying shrinkage and temperature shrinkage properties. Basler et al. [12] examined the effects of different vibration parameters on early-age concrete strength, finding that while vibration parameters have a limited direct effect on compressive strength, applying vibration before initial setting can markedly enhance strength. Bai et al. [13] used SEM to observe that concrete prepared by vibratory mixing exhibits a higher degree of hydration, fewer defects and pores, and a denser microstructure. Guan et al. [14] studied vibratory mixing of fiber-reinforced recycled aggregate concrete and found that vibration promotes uniform distribution of the mixture, thereby increasing compressive strength, splitting tensile strength, and flexural strength, respectively. Xiong et al. [15] prepared lightweight aggregate concrete using vibratory mixing and demonstrated that vibratory mixing facilitates the dispersion of high-dosage steel fibers, resulting in a more uniform fiber orientation compared with conventional mixing. Si et al. [16] investigated the influence of vibratory mixing on the properties of fluidized solidified soil and found that water stability is significantly enhanced under vibratory mixing.

Third, optimization studies on key parameters of vibratory mixing. Zhao et al. [17] investigated the influence of vibration acceleration (1 g, 2 g, 3.5 g) on the compressive strength and density of cement-stabilized macadam, finding that compressive strength increases with increasing vibration acceleration; however, at higher cement dosages, the enhancement effect of vibratory mixing is relatively limited. Mitrosz et al. [18] examined the effect of vibration duration on the porosity, permeability, and compressive strength of pervious concrete containing 50% recycled concrete aggregate, indicating that increasing vibration duration reduces porosity and permeability while enhancing compressive strength. Kou et al. [19] developed a flow field simulation model for the vibratory mixing process of cement paste, demonstrating that vibratory mixing significantly improves mixing effectiveness, with mixing efficiency and uniformity positively correlated with excitation frequency.

However, most existing studies treat vibration as a holistic “energy input,” focusing primarily on the improvement effects and parameter optimization of vibratory mixing, with few investigations approaching the problem from the fundamental perspective of mechanical wave propagation. To date, only Yue [20] has theoretically calculated the effective propagation distance of a vibratory exciter, reporting a 67% loss of vibration energy at a distance of 230 mm. However, given the higher viscosity of the material investigated in the present study, this theoretically derived value may not be directly applicable to steel fiber-reinforced concrete (SFRC).

Based on this research gap, this study systematically investigates the propagation patterns of vibration waves in fresh cement paste under vibratory mixing at different frequencies. The analysis focuses on the attenuation patterns of vibration intensity (VI) and particle velocity (PV) in SFRC under various water–binder ratios. Furthermore, from the perspective of wave energy conservation, the energy transfer during vibratory mixing is examined. The research findings aim to elucidate the propagation mechanisms of vibratory mixing, providing a theoretical foundation and scientific basis for optimizing vibratory mixing processes and developing efficient mixing equipment.

## 2. Materials and Methods

### 2.1. Raw Materials

As the most abundant cementitious material in low water–binder ratio SFRC, cement plays a crucial role in determining the mechanical properties and durability of concrete. In this experiment, Dongyue brand P.O52.5 ordinary Portland cement produced by Shandong Shanshui Cement Group Co., Ltd. (Jinan, China) was selected. The basic performance indicators are shown in Table 1, and the clinker composition is presented in Table 2. The fly ash, silica fume, and mineral powder were all produced by Henan Borun Foundry Material Co., Ltd. (Gongyi, China). The density of fly ash is 2.55 g/cm^3^. The density of silica fume is 2.2 g/cm^3^, with a specific surface area of 22.1 m^2^/g and a SiO_2_ content of >98%. The density of mineral powder is 2.9 g/cm^3^, with a specific surface area of 412 m^2^/kg. The composition of cementitious materials is detailed in Table 3. Basalt (Xuchang, China) was used as coarse aggregate, and quartz sand (Xuchang, China) was used as fine aggregate. The quartz sand was classified into medium-coarse and fine particle sizes, mixed in a 1:1 ratio. All material properties fully comply with the requirements of the “Test Methods of Cement and Concrete for Highway Engineering”.

The steel fiber used in this study was a copper-coated steel fiber produced by Jiangsu Sobute New Materials Co., Ltd. (Nanjing, China), with a density of 7.8 g/cm^3^, length of 13 mm, diameter of 0.2 mm, and tensile strength not less than 1200 MPa. The superplasticizer employed was a polycarboxylate-based high-performance water reducer manufactured by Jiangsu Sobute New Materials Co., Ltd., appearing as a light-yellow transparent liquid with a solid content of 30% and a water reduction rate of 40%.

The SFRC mix proportions adopted in this experiment are presented in Table 4. Two water–binder ratios, 0.2 and 0.16, were utilized, and vibratory mixing was conducted at frequencies of 30 Hz, 120 Hz, and 180 Hz. For each testing condition, three systems were designed for comparison: the reference group (Series J), the series without coarse aggregate (Series A), and the series incorporating 5–10 mm coarse aggregate (Series B).

### 2.2. Experimental Program

#### 2.2.1. Preliminary Test on Vibration Propagation Distance

**Figure 1:**
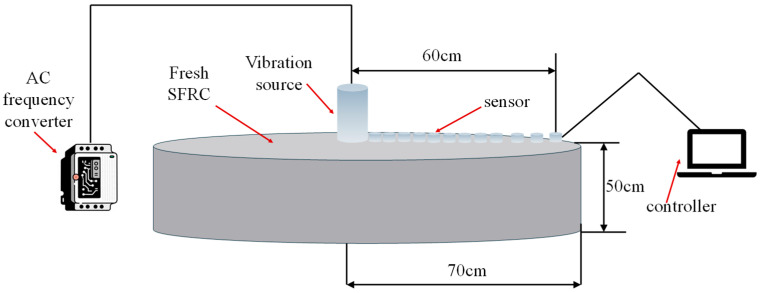
Schematic diagram of the experimental setup for testing mechanical vibration propagation distance in fresh SFRC.

The experimental apparatus used in this study consisted of a mechanical vibration source, a cylindrical cast iron cylinder (radius 70 cm, height 50 cm), an AC frequency converter, vibration acceleration sensors and PV sensors with corresponding testers (Figure 1), with the aim of macroscopically evaluating the propagation range of vibration at a relatively large scale. It should be noted that only one type of sensor—either acceleration or PV—could be deployed at a time; therefore, the sensor positions marked in Figure 1 represent the common measurement layout used sequentially for both types of sensors. A representative mix proportion (designation A4) of fresh SFRC was selected for testing. Tests were conducted at three frequencies—30 Hz, 120 Hz, and 180 Hz—and each test was repeated three times on samples from the same batch to assess repeatability. During testing, the sensors were arranged along the radial direction of the cylinder, extending from near the source outward to a maximum distance of 60 cm. Data acquisition at each measurement point commenced simultaneously only after the vibration source had reached stable operation within the fresh SFRC.

#### 2.2.2. Propagation Attenuation Test of Vibration

**Figure 2:**
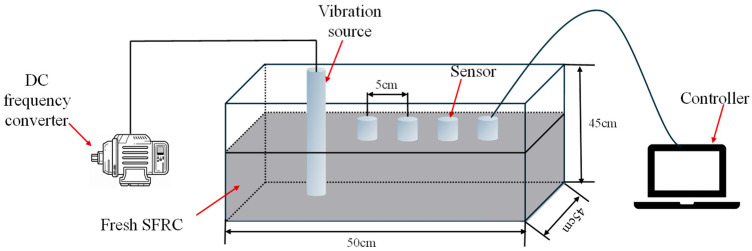
Schematic diagram of the experimental setup for testing mechanical vibration propagation in fresh SFRC.

Based on the results of the preliminary tests, attenuation tests were further conducted to accurately quantify the near-field attenuation behavior. The experimental apparatus for this test (Figure 2) replaced the iron cylinder with a transparent acrylic cube (50 × 45 × 45 cm) to facilitate visual observation, and the AC frequency converter was upgraded to a DC frequency converter with higher control precision. The core measurement region was confined within 20 cm from the vibration source, within which an array of integrated sensors for vibration acceleration and PV was arranged at intervals of 5 cm. It should be noted that only one type of sensor—either acceleration or PV—could be deployed at a time; therefore, the sensor positions marked in Figure 2 represent the common measurement layout used sequentially for both types of sensors. A full factorial experimental design was adopted in this study to systematically investigate the effects of two variables: water–binder ratio (0.20 and 0.16) and vibration frequency (30, 120, and 180 Hz). For each combination of water–binder ratio and frequency, three independently prepared replicate samples were tested to ensure statistical robustness of the results. In each test group, data from the sensor array were collected after the vibration had stabilized.

## 3. Conclusions and Analysis

### 3.1. Distance Attenuation Characteristics of Vibration Energy

The propagation characteristics of vibration in fresh SFRC are described by two core parameters: VI and PV. VI represents the amplitude of the vibration wave and characterizes the energy intensity transmitted to a given location, as defined by Equation (1):

(1) $$\mathrm{VI}\;=\frac{A\omega^{2}}{g}$$

where A is the amplitude, ω is the angular frequency, and g is the gravitational acceleration. PV reflects the motion speed of medium particles induced by the vibration wave, indicating the degree of mechanical disturbance. Together, these two parameters elucidate the mechanism of vibration wave action in viscoelastic media from the perspectives of energy and kinematics, respectively.

**Figure 3:**
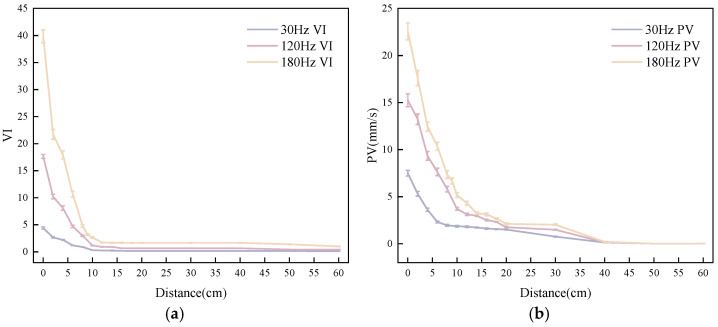
Attenuation curves of VI and PV with propagation distance in fresh SFRC: (**a**) VI; (**b**) PV.

As shown in Figure 3, both VI and PV exhibit exponential attenuation characteristics with a clear dependence on frequency. Higher frequencies correspond to shorter wavelengths, resulting in more rapid energy dissipation in the viscoelastic medium, which is manifested as steeper fitting curve slopes and earlier occurrence of attenuation inflection points. Within 10 cm from the vibration source, VI decreases approximately linearly to about 5% of its initial value. A gentle transition observed in the 2–4 cm range is attributed to the high VI near the source surface, where energy is rapidly consumed during the initial stage of propagation, while near 10 cm, increased medium resistance temporarily slows the attenuation rate. At a propagation distance of 20 cm, the VI curve stabilizes at a residual level of approximately 3–4%, and subsequent attenuation becomes negligible.

The attenuation trend of PV is generally similar to that of VI, although the absolute attenuation at a given distance is smaller, indicating a relatively better propagation capability. Nevertheless, the rate of change in PV also approaches zero at 20 cm. The convergence of both parameters toward a near-zero attenuation rate at 20 cm mutually confirms that, under the present experimental conditions, the effective propagation distance of vibration in fresh SFRC is approximately 20 cm from the vibration source.

In summary, under the conditions of this study, the effective propagation distance of vibration waves is approximately 20 cm from the vibration source.

### 3.2. Spatial Distribution Patterns

**Figure 4:**
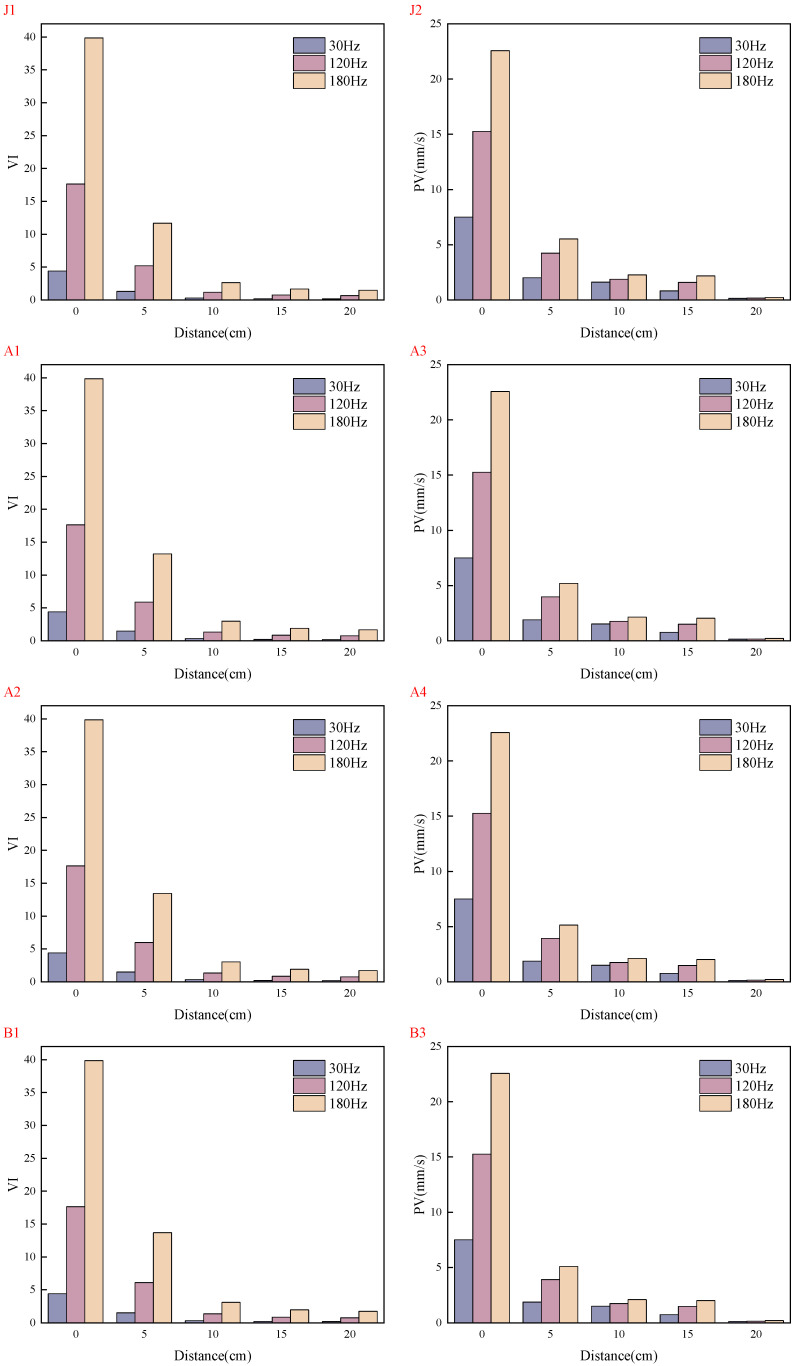

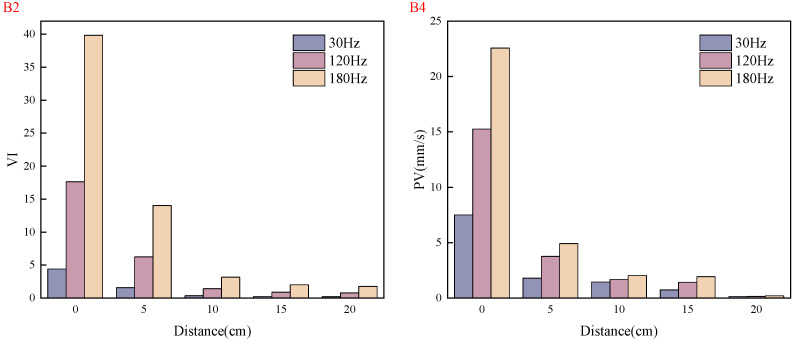
Comparison of VI under different working conditions.

**Figure 5:**
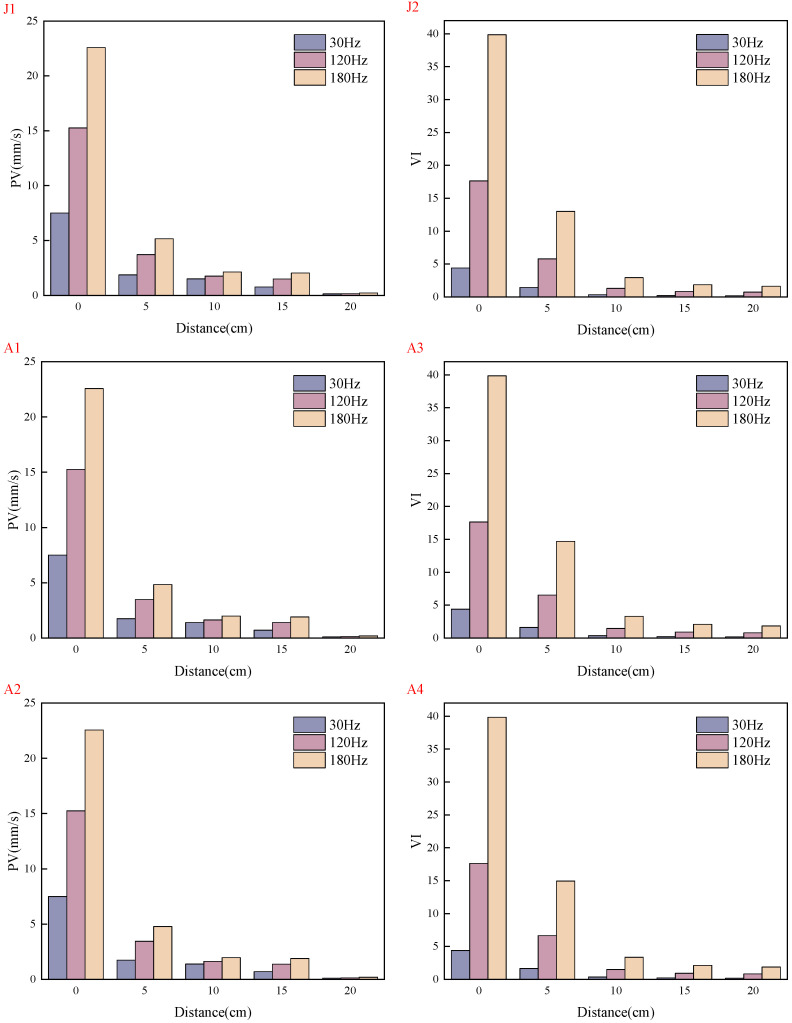

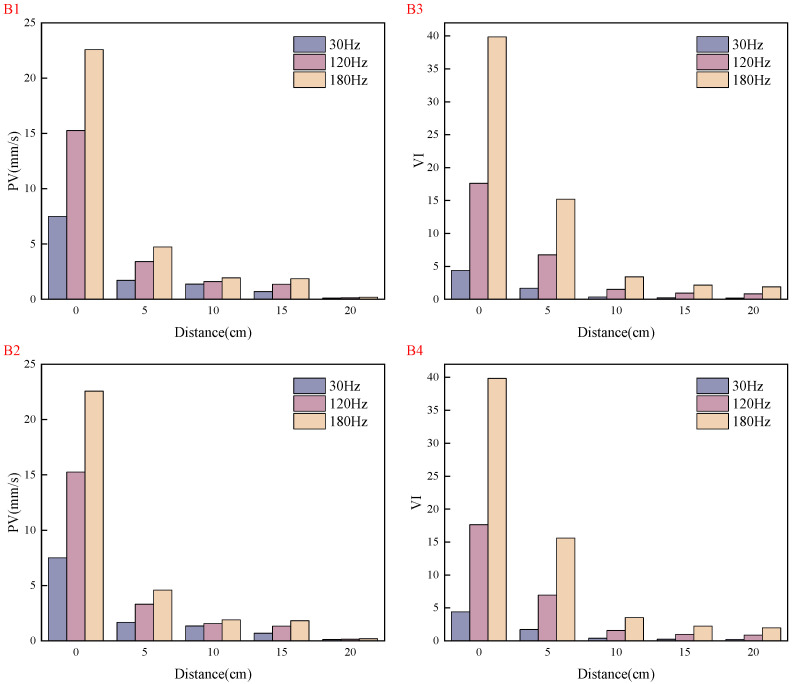
Comparison of PV under different working conditions.

Under the conditions of water–binder ratios of 0.2 and 0.16, the distributions of VI and PV across five mix proportions are shown in Figure 4 and Figure 5, and the data in the figures are the average values from three parallel tests.

Both VI and PV exhibit rapid attenuation with increasing propagation distance, with VI attenuating at a faster rate than PV. At 5 cm from the vibration source, VI attenuates to 50% of its initial value; at 10 cm, it attenuates to approximately 10%; and at 20 cm, it attenuates to less than 3%. PV shows relatively smaller attenuation at corresponding distances: approximately 60% at 5 cm, approximately 30% at 10 cm, and attenuating to less than 3% at 20 cm.

The vibration attenuation behavior is governed by the combined effects of vibration frequency and material mix proportion. Vibration frequency is a core factor influencing attenuation. Studies indicate that medium and high frequencies (120 Hz and 180 Hz) exhibit more pronounced attenuation characteristics compared with low frequency (30 Hz), with the measured values of vibration intensity and PV increasing quadratically with frequency. Material mix proportion also significantly modulates vibration propagation. First, a reduction in the water–binder ratio (to 0.16) enhances the cohesiveness and density of the system, resulting in an approximately 10% decrease in peak vibration intensity and an approximately 10% increase in peak PV, while both parameters exhibit relatively slower attenuation rates under the lower water–binder ratio. Second, the incorporation of fiber creates an interlocking three-dimensional network structure, transforming the fresh mixture into a “fiber-reinforced viscoelastic solid.” This substantially increases yield stress and plastic viscosity and introduces strong anisotropy, collectively contributing to an additional attenuation of vibration intensity by approximately 10%. Furthermore, the addition of coarse aggregate dissipates energy through mechanisms such as increased solid volume fraction, interparticle friction, and interfacial scattering; however, under the present experimental conditions, the resulting attenuation amplitude of vibration intensity (approximately 4%) is smaller than that observed in the fiber-incorporated groups.

To systematically reveal the propagation mechanism and attenuation patterns of mechanical vibration in fresh SFRC, an exponential attenuation model was established in this study based on sensor measurement data. Numerical simulations of the two-dimensional spatial distribution of vibration acceleration intensity and particle PV were conducted using the MATLAB(2022b) software platform. Through spatial interpolation and grid calculation, the simulation results achieved a visual representation of the spatial attenuation characteristics of vibration energy in fresh SFRC. Furthermore, by uniformly fixing the color axis range and color mapping rules, a consistent correspondence between colors and the numerical values of VI and PV was ensured, thereby enabling comparability of the color-value mapping relationships across different images.

**Figure 6:**
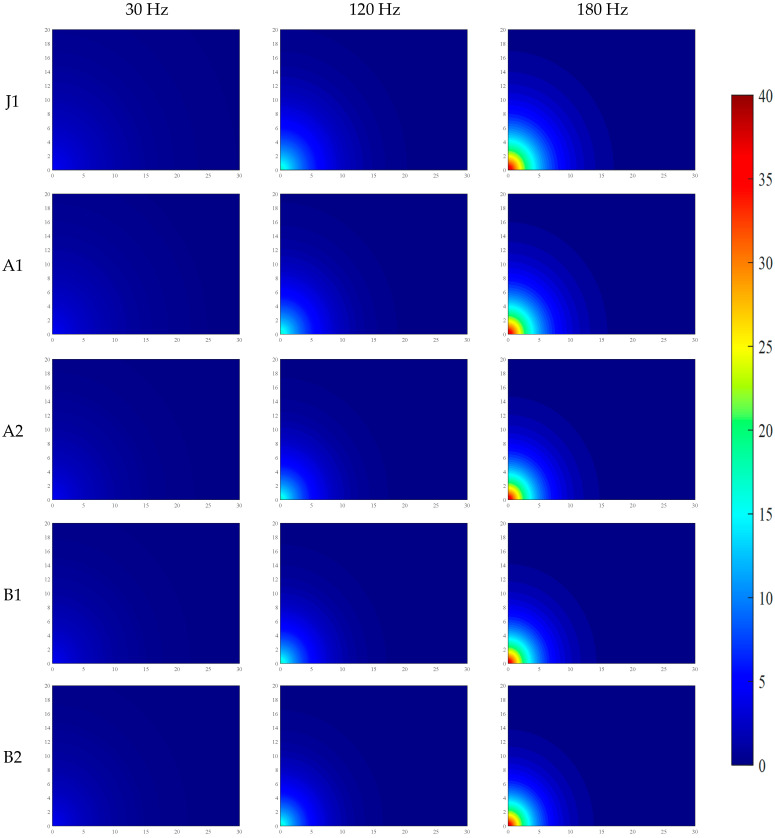
Distribution of VI in fresh SFRC with a water–binder ratio of 0.2.

**Figure 7:**
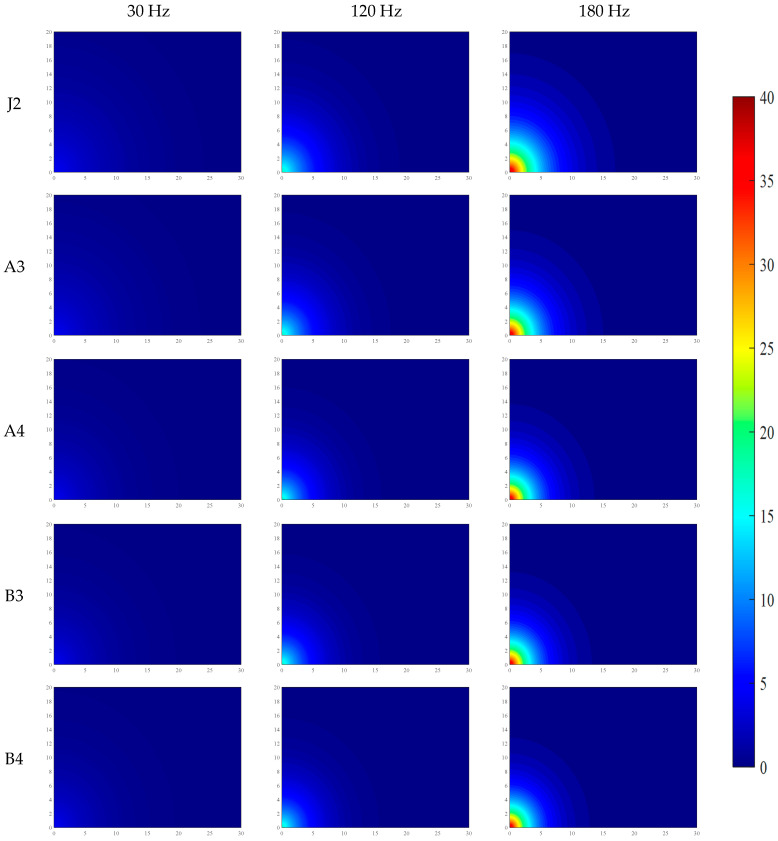
Distribution of VI in fresh SFRC with a water–binder ratio of 0.16.

**Figure 8:**
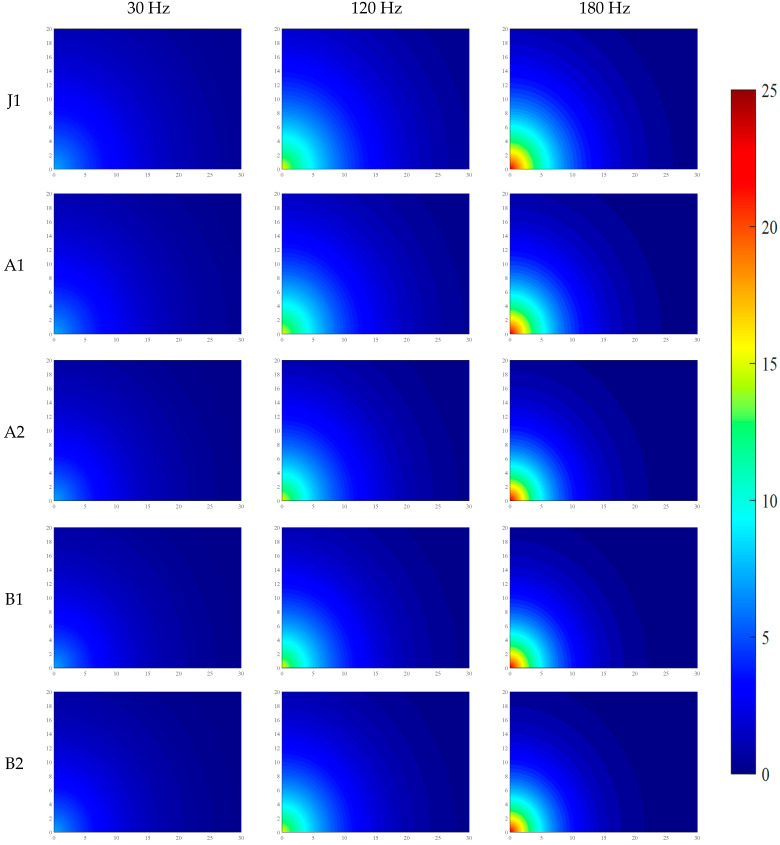
Distribution of PV in fresh SFRC with a water–binder ratio of 0.2.

**Figure 9:**
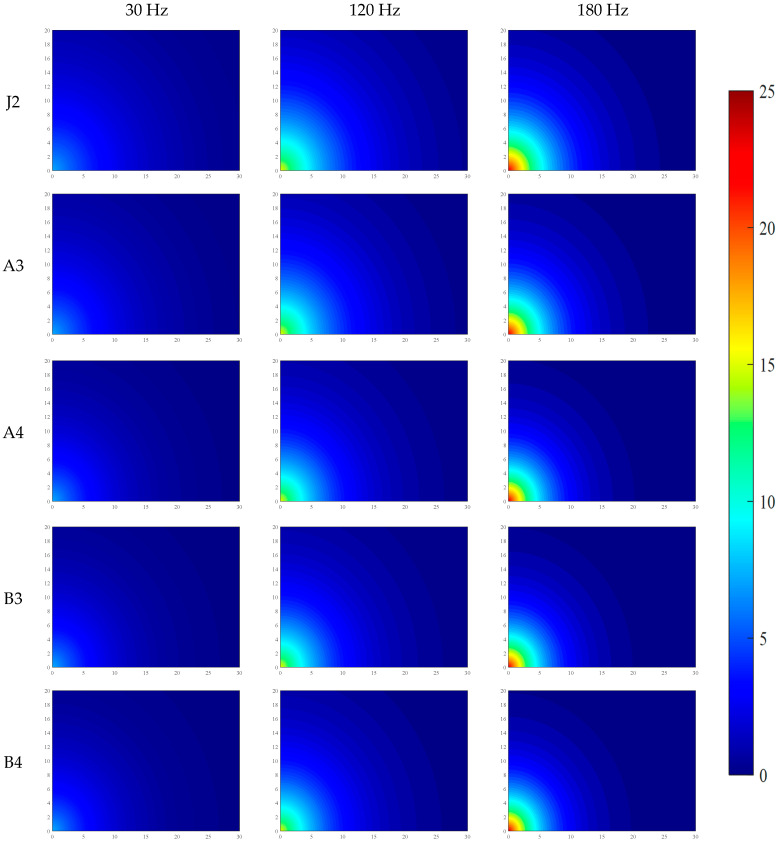
Distribution of PV in fresh SFRC with a water–binder ratio of 0.16.

The relevant simulation results have been systematically compiled in Figure 6, Figure 7, Figure 8 and Figure 9, providing a basis for the quantitative analysis and optimization of vibration processes.

As shown in Figure 6, Figure 7, Figure 8 and Figure 9, the propagation of mechanical vibration in fresh SFRC exhibits significant attenuation characteristics: both vibration intensity and PV decrease rapidly with increasing propagation distance. Notable differences in attenuation behavior are observed under different frequencies. Under the low-frequency condition of 30 Hz, although the input energy is relatively low, the vibration propagates over a comparatively longer distance. In contrast, under the medium frequency of 120 Hz and the high frequency of 180 Hz, despite higher near-field initial vibration intensity, the energy attenuates more sharply along the radial direction. Taking the J1 mix with a water-to-binder ratio of 0.2 as an example, under the 30 Hz condition, the vibration intensity at 10 cm from the vibration source remains approximately 22% of the central region intensity, and retains about 3% residual intensity at 30 cm. By comparison, under the 120 Hz and 180 Hz conditions, the intensity at 10 cm drops to approximately 19% of the central value, and generally decays to around 1% at 30 cm. The spatial distribution of PV exhibits a similar trend: under the 30 Hz condition, the PV decreases gradually along the radial direction, whereas under the 120 Hz and 180 Hz conditions, a steep decline occurs within the 15–20 cm range from the vibration source.

Furthermore, a comparison across different mix groups reveals that increasing fiber and aggregate content enhances the propagation of vibration intensity yet exerts a slight retarding effect on the propagation of PV. The water-to-binder ratio also has a significant influence on vibration propagation characteristics. Compared with the 0.20 water-to-binder ratio system, the 0.16 system exhibits stronger attenuation effects on both vibration intensity and PV, as manifested by the broader distribution of low-value blue regions in the figures.

## 4. Energy Conservation Model

Since SFRC can serve as a continuous medium for mechanical vibration propagation, and SFRC under low water–binder ratio conditions behaves as a viscoelastic body, traditional models such as the Kelvin–Voigt model and gradient theory models struggle to accurately describe the propagation behavior of mechanical vibrations in fresh mixtures [21]. The key challenge lies in the fact that multiple parameters involved in these models—such as the time-dependent elastic modulus E(t) and viscosity coefficient η(t), as well as the microstructural parameters g and h in gradient elasticity theory—are difficult to measure directly and are subject to significant errors during experimental measurement [22]. Furthermore, mechanisms such as the distribution of vibration energy upon entering the material and factors like mechanical damping are also challenging to quantify precisely, further increasing the difficulty of theoretical modeling.

Based on the above considerations, this study proposes a simplified analytical method focused on macroscopic energy balance, building upon the experimental measurements and simulations presented in Section 3.2. The core of this method is to neglect the complex details of energy dissipation along the propagation path and instead verify whether the vibration propagation process adheres to the principle of energy conservation by integrating the vibration input work and the work absorbed by the medium. To ensure the simplicity and feasibility of the model, the following key simplifying assumptions were established prior to model formulation, and their justification is discussed below:

1. The influence of the shear wave (S wave) and surface waves is neglected. In a predominantly viscous medium such as fresh concrete, longitudinal waves serve as the primary carrier of vibration energy over long distances. S waves and surface waves exhibit slower wave speeds and extremely rapid attenuation, with their effective influence typically confined to within a few wavelengths of the vibration source. Therefore, within the primary region of interest, S-wave and surface-wave contributions are disregarded, and the analysis focuses on longitudinal wave energy transfer.
2. The PV is assumed to be uniform across any given radial cross-section. This assumption reduces the three-dimensional problem to an axisymmetric model, thereby facilitating analytical solutions.
3. Significant cumulative thermal effects induced by vibration are neglected. Under the short vibration duration and open experimental conditions employed in this study, the input vibration energy is unlikely to be converted into sensible heat of the system due to the “vibrational cooling” effect of the material.

Based on the above analysis and assumptions, the energy expression at a specific particle location within the fresh SFRC is established, as presented in Equation (2).

(2) $$\int_{t_{1}}^{t_{i}}(p_{t}-p_{j})dt=\int {v_{l}}^{2}dm+m_{l}\cdot c\cdot \nabla T$$

where $p_{j}$ represents the vibration power of the vibration source under no-load conditions; $p_{t}$ represents the vibration power of the mechanical vibration at a distance *t* from the source in fresh SFRC; $v_{l}$ represents the acceleration of a particle at a distance *l* from the vibration source in fresh SFRC, which can be calculated as the product of VI and the gravitational acceleration *g*; $m_{l}$ represents the mass of a particle at a distance *l* from the vibration source in fresh SFRC; *c* represents the specific heat capacity of the SFRC; and ∇T represents the temperature rise in the particle.

To obtain the vibration power data of the source, the vibration source was started first during the mixing process. Fresh SFRC was rapidly added at 3 s of operation, and subsequent power variations were continuously measured.

**Figure 10:**
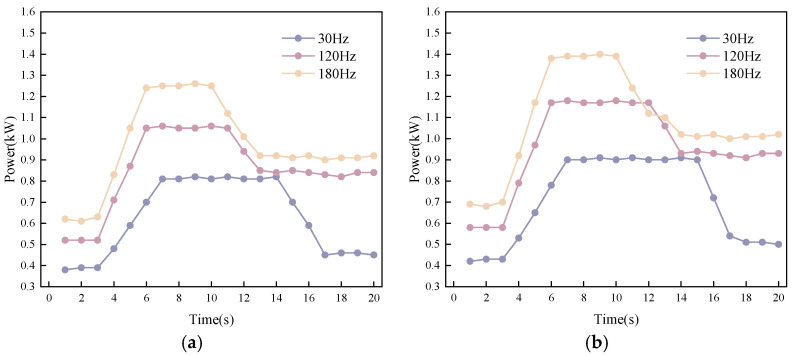
Vibration power variation: (**a**) A2; (**b**) B4.

As shown in Figure 10, the vibration power increases rapidly after being transmitted into the fresh SFRC and stabilizes after approximately 3–4 s, with the stable value reaching more than twice that under no-load conditions. At this point, the PV also enters a stable state. Subsequently, the power decreases, which can be attributed to local liquefaction of the material reducing the resistance force, thereby lowering the required input power accordingly. The system then reaches a new equilibrium state. The power variation process provides data support for the integral calculation of power differences in Equation (2).

The PV can be obtained from Figure 8 and Figure 9. Based on these assumptions, the vibration response of the unit mass system can be expressed as Equation (3), and the PV expression is shown as Equation (4). The particle PV at distance *l* from the vibration source has been numerically verified in the MATLAB simulation.

(3) $$dm=\rho L\pi {\left(dl\right)}^{2}$$

(4) $$V=Ae^{-Bl}$$

**Figure 11:**
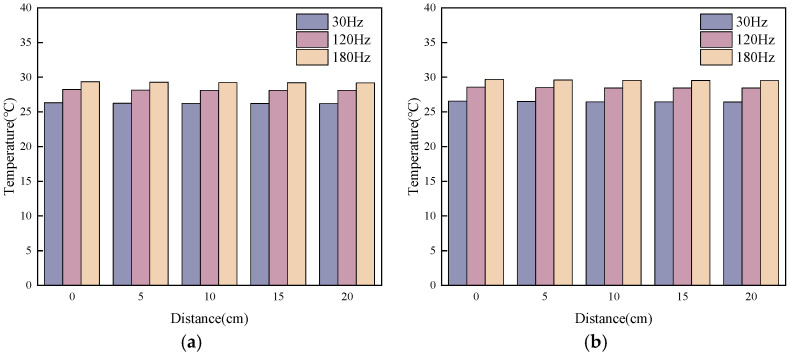
Temperature variation in SFRC under vibration: (**a**) A2; (**b**) B4.

The temperature rise in fresh SFRC induced by mechanical vibration can be directly measured using a thermal imaging camera. Measurements revealed that within 20 s of vibration application, temperature readings obtained using precision thermocouples in the fresh SFRC remained essentially stable. This phenomenon is primarily attributed to the “vibration cooling” effect resulting from increased evaporation and convective diffusion caused by vibration. Under open experimental conditions, the temperature rise in fresh SFRC due to vibrational heat generation was on the order of 0.05 K. Specific test data measured with a Pt100 high-precision Class A platinum resistance sensor appear in Figure 11. It should be noted that accurate measurement under these open conditions was challenging.

Therefore, the energy conservation equation can be expressed as Equation (5).

(5) $$\int_{t_{i}}^{t_{j}}(p_{t}-p_{j})dt=\int_{l_{i}}^{l_{2}}A(e^{-Bl}{)}^{2}\rho L\pi (dl{)}^{2}+m_{l}\cdot c\cdot \nabla T$$

where *ρ* represents the density of the SFRC, *L* represents the unit depth of the vibration source, and *l* represents the distance between the particle and the vibration source.

To quantitatively evaluate the validity of the three key simplifying assumptions adopted in this study and their impact on the final conclusions, a complete error propagation verification case was constructed. The objective of this case is to quantify and synthesize the uncertainties introduced by the assumptions and to demonstrate that, within the combined error range, the core conclusion of the model remains valid, thereby confirming the robustness of the model.

The uncertainty of the model primarily originates from the three fundamental assumptions stated above. Taking the A2 mix proportion under the 30 Hz condition as a representative case, the relative errors introduced by each neglected term and their synthesized total uncertainty are presented in Table 5. The synthesized uncertainty of ±5.9% is considerably smaller than the acceptable deviation range for energy balance verification, indicating that the model simplifications do not obscure the macroscopic trend of energy conservation, thereby substantiating the robustness of the proposed analytical approach.

Before the SFRC yields and reaches equilibrium, the vibration input energy is primarily consumed in overcoming internal resistance within the material, with relatively little energy converted into heat. After the power balance is achieved, the energy is mainly dissipated as heat. Therefore, in the process of verifying energy conservation, a stage-by-stage analysis is more reasonable.

**Figure 12:**
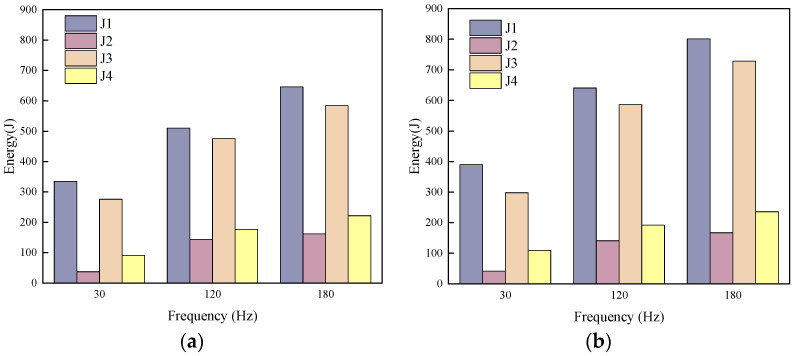
Energy conservation verification: (**a**) A2; (**b**) B4.

Taking the two mixture proportions A2 and B4 as research subjects, the entire vibration process and the corresponding energy response of the SFRC were verified, with the results shown in Figure 12. The input energy during vibration propagation was defined as the vibration work stage J_1_ and the heat conversion stage J_2_, while the effective energy received by the SFRC was defined as the vibration work stage J_3_ and the heat conversion stage J_4_. The verification results indicate that under different vibration frequency conditions, J_1_ + J_2_ ≈ J_3_ + J_4_ is satisfied, demonstrating that the energy propagation of mechanical vibration in SFRC conforms to the law of energy conservation.

## 5. Results and Discussion

(1) The propagation distance of mechanical vibration in SFRC with a low water–binder ratio exhibits an exponential attenuation characteristic. Although some scholars have drawn an analogy between mechanical vibration propagation in concrete and seismic wave propagation and have proposed corresponding empirical formulas for propagation distance, the applicability of these existing formulas remains limited due to significant differences in cohesiveness and viscous resistance among various concrete systems. The present study, focusing on SFRC systems with low water–binder ratios, provides preliminary insights into the propagation attenuation behavior of mechanical vibration in such materials.
(2) The spatial distribution characteristics of VI and PV in fresh SFRC indicate that both parameters are closely related to vibration frequency and mixture proportion parameters. Through a combination of experimental measurements and MATLAB numerical simulation, this study systematically analyzed the distribution patterns of the parameters in the paste. Currently, research on the propagation characteristics of vibration in concrete is relatively scarce. Based on sensor testing and data analysis methods, this study has preliminarily explored the propagation behavior of VI and PV in fresh SFRC.
(3) Based on the principle of energy conservation, a balanced relationship was established between the vibration input energy and the kinetic and thermal energy obtained by the concrete system. Through simplified testing and preliminary verification, it was found that the energy transfer process during the introduction of vibration in SFRC mixing can be described by the energy conservation equation. It should be noted that this study only performed energy conservation verification at selected nodes; systematic experimental research is still needed in the future to comprehensively reveal the evolution and distribution patterns of vibration energy throughout the entire mixing process.
(4) The vibration propagation characteristics revealed in this study provide direct guidance for the engineering design of vibratory mixing equipment. Based on the experimental finding that the vibration wave attenuates significantly at 20 cm, it is recommended that the spacing between vibrators should not exceed 40 cm to ensure overlapping coverage of the effective action zones and thereby achieve uniform treatment of the material within the mixing chamber. Regarding the selection of operating frequency, given that low-frequency (30 Hz) vibration exhibits a longer propagation distance, whereas medium and high frequencies (>120 Hz) undergo rapid attenuation, a medium-to-low frequency range of 30–80 Hz is recommended. This range offers a balance between energy penetration depth and local shear intensity, thereby optimizing fiber dispersion and overall fluidization effects. Furthermore, for the highly viscoelastic medium of SFRC with a low water–binder ratio, the driving power of the vibratory mixer should be appropriately increased to ensure that sufficient vibration intensity is delivered at the periphery of the effective action radius to overcome interparticle cohesion and promote uniform mixing.

## 6. Conclusions

Based on the experimental results and analysis, the following conclusions can be drawn:

(1) The propagation of mechanical vibration in low water–binder ratio steel fiber-reinforced SFRC exhibits significant spatial attenuation characteristics, with an effective propagation distance of approximately 20 cm from the vibration source. Within this range, the attenuation behavior of the vibration parameters is clearly defined, providing a basis for determining the appropriate testing range.
(2) The attenuation behavior of vibration intensity and displacement velocity is primarily governed by vibration frequency, with medium and high frequencies (120 Hz and 180 Hz) exhibiting substantially more pronounced attenuation than low frequency (30 Hz). Additionally, among the material mix proportions, a lower water–binder ratio tends to slow the attenuation rate, whereas the incorporation of fibers and coarse aggregate exacerbates attenuation to varying degrees.
(3) The influence of material modification on vibration propagation varies in magnitude. The incorporation of fibers results in an additional attenuation of approximately 10%, a phenomenon primarily attributed to the three-dimensional network structure formed by the fibers. In contrast, the influence of coarse aggregate is relatively smaller, contributing to an attenuation of approximately 4%.
(4) Based on the principle of energy conservation, an equation describing the transfer of vibration energy during the mixing process was preliminarily established and experimentally validated, thereby providing a theoretical foundation for the quantitative analysis of vibration energy dissipation.

## Figures and Tables

**Table 1:** Basic physical and mechanical properties of cement.

Apparent Density (g/cm^3^)	Initial Setting Time (min)	Final Setting Time (min)	Standard Consistency (%)	Stability (mm)	Loss on Ignition (%)	Flexural Strength (MPa)	Compressive Strength (MPa)
3.12	182	248	28.6	Qualified	3.79	9.3	61.2

**Table 2:** Mineral composition of cement.

Composition	C_3_S	C_2_S	C_4_AF	C_3_A
Content (%)	59.5	18.1	9.9	7.4

**Table 3:** Main chemical compositions of cementitious materials (%).

Materials	CaO	SiO_2_	Al_2_O_3_	MgO	SO_3_	Fe_2_O_3_	Others
Cement	68.42	18.27	0.64	4.44	2.9	2.48	2.85
Fly ash	7.60	47.1	34.20	0.86	2.10	3.85	4.29
Silica fume	0.36	98.1	0.11	0.32	0.86	0.10	0.15
Mineral powder	39.29	33.06	15.04	9.96	1.90	0.30	0.45

**Table 4:** SFRC experimental mix proportions (kg/m^3^).

Test Condition	Water–Binder Ratio	Grouping	Binder Material	Quartz Sand	Water	Superplasticizer	Steel Fiber	Coarse Aggregate
Comparison of mixing conditions under different vibration frequencies of 30 Hz, 120 Hz and 180 Hz	0.2	J1	1085	905	217	11.07	/	/
		A1		905		11.07	78	/
		A2		905		11.07	156	/
		B1		814		11.07	156	91
		B2		724		11.07	156	181
	0.16	J2	1085	905	173.6	11.07	/	/
		A3		905		11.95	78	/
		A4		905		11.95	156	/
		B3		814		11.95	156	91
		B4		724		11.95	156	181

**Table 5:** Relative errors introduced by model simplifications and synthesized uncertainty.

Error Source	Quantification Basis	Relative Error
Neglect of S wave	Based on the viscoelastic wave attenuation model, residual shear wave energy ≤ 3%	±3%
Assumption of uniform PV	Based on the spatial statistics of PV (Figure 8), CV ≈ 3.5%	±5%
Neglect of thermal accumulation	Based on measured ΔT (Figure 11); thermal contribution ≤ 1%	±1%
Synthesized uncertainty	Root Sum Square	±5.9%

## Data Availability

The original contributions presented in this study are included in the article. Further inquiries can be directed to the corresponding author.

## Abbreviations

The following abbreviations are used in this manuscript:

PV	particle velocity
VI	vibration intensity
SFRC	steel fiber-reinforced concrete
UHPC	Ultra-High Performance Concrete
S wave	Shear wave
CV	coefficient of variation

## References

[1] Ni M., Fan L. (2025). Intelligent Measurement and Control System for Workability of Fresh Concrete. Constr. Mach. Equip..

[2] Kaiyin Z. (2022). Study on the Power Characteristics of Vibration and Multi-Step Mixing Concrete and Its Change Rules. Ph.D. Thesis.

[3] Ying W. (2019). Study on Mixing Characteristics of Double Shaft Concrete Mixer Blades. Master’s Thesis.

[4] Zheng Y. (2020). Simulation Research of Double Horizontal Axis Concretemixer Used in Experiment. Master’s Thesis.

[5] Liu L. (2018). Research on the Mixing Performance for the Double Horizontal Ribbon Mixer on theDiscrete Element Method. Master’s Thesis.

[6] Zhao M., Liu H., Wang Q., Wang M., Chen Z., Wang X. (2025). Performance enhancement and mechanism analysis of low-carbon cement-stabilized macadam based on vibratory mixing method. Case Stud. Constr. Mater..

[7] Tian M., Xiong C., Chen W., Zhou Y., Liu B. (2022). Mixing homogeneity of cement-stabilized macadam and its influence mechanism on road performance. Vibroeng. Procedia.

[8] Zheng Y., Zhou Y., Huang X., Min Y., Luo H., Chen Y., Li W. (2022). Study on performance improvement of ultra-high performance concrete by vibration mixing. Constr. Build. Mater..

[9] Zhang F., Wei B., Li X., Zhang H. (2022). Study on Mechanical Properties of UHPC Based on Vibration Mixing Technology. Constr. Technol..

[10] Li X., Zhou Y., Xu J., Ning Z., Diab A. (2023). Macro- and Microscale Properties of Vibration Mixing Cement-Stabilized Macadam: Laboratory and Field Investigation. Iran. J. Sci. Technol. Trans. Civ. Eng..

[11] Wang Y., Zhang J., Wang X., Zhang Z. (2020). Laboratory Investigation on the Performance of Cement Stabilized Recycled Aggregate with the Vibration Mixing Process. Math. Probl. Eng..

[12] Basler F., Mahner D., Fischer O., Hilbig H. (2023). Influence of early-age vibration on concrete strength. Struct. Concr..

[13] Bai F., Wang X., Yang F., Duan K. (2024). Research on the Mechanical Properties of C50 Concrete under the Optimization of Vibration Mixing Process. Highway.

[14] Guan T., Zhang L., Zheng Y., Guan J., Zhang Y., Zhang Y. (2024). Frost resistance and damage evolution model of basalt fiber-reinforced recycled concrete based on recycled coarse aggregate strengthening and vibration mixing processes. J. Build. Eng..

[15] Xiong G., Wang C., Zhou S., Zheng Y. (2022). Study on dispersion uniformity and performance improvement of steel fibre reinforced lightweight aggregate concrete by vibrational mixing. Case Stud. Constr. Mater..

[16] Si Y., Wang W., Cao F., Li T., Zhang L., Lv X. (2022). Study on Properties of Cement Stabilized Macadam with Recycled Aggregate of Different Replacement Rates under Vibrating Mixing. J. Phys. Conf. Ser..

[17] Zhao K., Zhao L., Hou J., Feng Z., Jiang W. (2022). Impact of Mixing Methods and Cement Dosage on Unconfined Compressive Strength of Cement-Stabilized Macadam. Int. J. Concr. Struct. Mater..

[18] Mitrosz O., Kurpinska M., Miskiewicz M., Brzozowski T. (2025). Effect of vibration duration on strength and permeability of pervious concrete with recycled aggregate and low-carbon cements. Sci. Rep..

[19] Kou Z.M., Tan T.Y., Cui H.T. (2025). Study on the Behavior of Cement Slurry Prepared by Vibration Agitation and Numerical Simulation of Flow Field. Buildings.

[20] Yue Y. (2009). Study on the Concrete Vibratory Mixing. Master’s Thesis.

[21] Cao G., Bai Y., Shi Y., Li Z., Deng D., Jiang S., Xie S., Wang H. (2024). Investigation of vibration on rheological behavior of fresh concrete using CFD-DEM coupling method. Constr. Build. Mater..

[22] McCraw M.R., Uluutku B., Solomon H.D., Anderson M.S., Sarkar K., Solares S.D. (2023). Optimizing the accuracy of viscoelastic characterization with AFM force-distance experiments in the time and frequency domains. Soft Matter.

